# Toward Personalized ACS Therapy: How Disease Status and Patient Lifestyle Shape the Molecular Signature of Autologous Conditioned Serum

**DOI:** 10.3390/jcm15031014

**Published:** 2026-01-27

**Authors:** Christoph Bauer, Daniela Kern, Kalojan Petkin, Stefan Nehrer

**Affiliations:** 1 Center for Regenerative Medicine, Department for Health Sciences, Medicine and Research, University for Continuing Education, 3500 Krems, Austria; daniela.kern@donau-uni.ac.at (D.K.); stefan.nehrer@donau-uni.ac.at (S.N.); 2 Center for Regenerative Medicine of the Musculoskeletal System, 4040 Linz, Austria; info@drpetkin.at

**Keywords:** osteoarthritis, autologous conditioned serum (ACS), cytokines, growth factors, personalized medicine

## Abstract

**Background/Objectives**: Autologous conditioned serum (ACS) is an intra-articular orthobiologic for osteoarthritis (OA) intended to shift the joint cytokine milieu toward an anti-inflammatory, pro-regenerative profile. In the present study, we compared the molecular composition of ACS (specifically IMPACT^®^ ACS) from OA patients with that of healthy controls and assessed demographic and lifestyle influences on mediator levels. **Methods**: ACS was prepared from the whole blood of 50 OA patients and 20 healthy controls using the IMPACT^®^ centrifugation system (Plasmaconcept, Cologne, Germany) with glass-bead incubation and standardized handling. Cytokines, growth factors, and matrix metalloproteinases (MMPs) were quantified using multiplex immunoassays and ELISA. To account for demographic imbalances across cohorts, the primary findings were verified using age- and sex-adjusted multiple linear regression models. **Results**: Pro-inflammatory mediators were minimal in both cohorts, with IL-1β undetectable and IL-6 and TNF-α at very low levels. IL-1 receptor antagonist (IL-1RA) was consistently present. Notably, OA-derived ACS exhibited a catabolic shift compared to controls, characterized by significantly higher MMP-2 and MMP-3 levels. Growth factor profiling showed lower TGF-β1 and TGF-β3 in OA-derived ACS, with TGF-β2 showing no significant difference after adjustment. Exploratory stratified analyses indicated potential differences across sex, BMI, smoking status, and diet for select mediators, though subgroup sizes were limited. **Conclusions**: ACS prepared with a standardized IMPACT^®^ protocol displays a broad anti-inflammatory profile. However, increased MMPs and isoform-specific differences in TGF-β reflect a disease-associated molecular imprint. Consequently, patient-related heterogeneity supports the need for standardized reporting and motivates further research into stratified ACS therapy.

## 1. Introduction

Osteoarthritis is one of the most prevalent chronic joint disorders, characterized by the progressive degeneration of articular cartilage, subchondral bone remodeling, and osteophyte formation. This degenerative cascade is further exacerbated by synovial inflammation and chronic joint pain, leading to functional impairment and reduced quality of life [1]. OA affects hundreds of millions of individuals worldwide, with its prevalence expected to rise further because of population ageing, increasing obesity rates, and a growing burden of joint injuries [1,2]. In addition, OA imposes a significant socioeconomic burden, including healthcare costs and loss of productivity [3].

Despite extensive research efforts, no curative treatment for OA is currently available. Conventional therapeutic strategies, including nonsteroidal anti-inflammatory drugs (NSAIDs), corticosteroid injections, and hyaluronic acid supplementation, predominantly focus on symptomatic pain relief and often fail to address the underlying inflammatory and degenerative processes within the joint [1]. This limitation underscores the urgent need for novel, disease-modifying approaches targeting the pathophysiological mechanisms driving OA progression.

A key factor in OA pathogenesis is the imbalance of cytokine signaling within the joint microenvironment. Elevated levels of proinflammatory cytokines such as interleukin-1β (IL-1β) and tumor necrosis factor-α (TNF-α) stimulate the production of matrix-degrading enzymes, notably matrix metalloproteinases (MMPs), which contribute to cartilage destruction and perpetuate inflammation [4,5]. In contrast, anti-inflammatory cytokines such as interleukin-1 receptor antagonist (IL-1RA) are present at insufficient levels in OA joints, limiting the natural counter-regulatory mechanisms necessary to inhibit tissue degradation [4,6]. This dysregulation promotes a self-amplifying cycle of inflammation, cartilage loss, and pain. As a result, there is increasing interest in therapeutic strategies that modulate the intra-articular cytokine milieu, shifting the treatment paradigm from symptomatic control toward potential disease modification [7,8].

Autologous conditioned serum (ACS) has emerged as an important therapeutic option. In contrast to platelet-rich plasma (PRP), which predominantly reflects platelet-derived mediators, ACS is generated from whole blood and therefore captures signals from leukocytes and other blood components in addition to platelets. Unlike cell-based injections such as mesenchymal stromal cell therapies, ACS is a purely cell-free serum product, which may offer advantages with respect to regulatory requirements and safety while still targeting key inflammatory pathways [9,10]. Developed in the mid-1990s and subsequently refined for various applications, ACS is generated by incubating whole blood with glass beads, thereby stimulating the release of a broad spectrum of bioactive molecules [11]. The resulting serum is enriched not only in anti-inflammatory cytokines such as IL-1RA and IL-4 but also in growth factors like transforming growth factor-β (TGF-β) and platelet-derived growth factor (PDGF), alongside lower concentrations of proinflammatory mediators, including IL-1β and TNF-α [7,10,12]. Following centrifugation, this biologically active serum is re-injected into the affected joint, targeting inflammation at the molecular level by competitively blocking IL-1 receptor binding. This mechanism enables ACS to reduce intra-articular inflammation, alleviate pain, and potentially promote tissue repair and regeneration. Consequently, ACS represents a more comprehensive strategy for OA management compared with conventional symptomatic treatments such as NSAIDs, corticosteroid injections, and intra-articular hyaluronic acid, which primarily target pain rather than the underlying inflammatory cascade [13,14].

Initial evidence from both veterinary and human medicine supports the therapeutic potential of ACS. In animal models, ACS has been shown to reduce clinical symptoms and improve joint mobility in horses and dogs with OA [14,15]. In human clinical trials, ACS has demonstrated significant improvements in pain relief and joint function, particularly in knee OA, with efficacy comparable or superior to platelet-rich plasma (PRP) and corticosteroids [6,7,8,16]. Importantly, ACS may exert disease-modifying effects by directly modulating inflammatory pathways, potentially slowing OA progression rather than merely addressing symptoms. However, several factors influence therapeutic outcomes, including variability in cytokine and growth factor concentrations depending on incubation times and patient-specific factors such as disease stage and individual responsiveness [8,10]. Therefore, standardized production protocols and a better understanding of influencing factors are crucial to optimizing the clinical application of ACS [7,10].

This study aimed to investigate differences in the composition of pro- and anti-inflammatory cytokines, growth factors, and degradative enzymes in autologous conditioned serum (ACS) derived from OA patients compared to healthy subjects. A significant knowledge gap remains regarding how specific lifestyle and demographic factors modulate the molecular profile of ACS, particularly when using standardized, short-incubation protocols. Furthermore, we evaluated questionnaire data to identify demographic and lifestyle factors that may influence ACS composition, thereby providing exploratory insights to potentially optimize and stratify ACS-based treatment strategies for OA.

We hypothesized that (i) ACS derived from OA patients would display a distinct molecular profile compared with ACS from healthy individuals, characterized by higher levels of degradative enzymes and altered growth factor concentrations; and (ii) demographic and lifestyle factors such as sex, BMI, smoking status, diet, and age would significantly modulate the molecular composition of ACS. Understanding these patterns was expected to provide a rationale for a more stratified use of ACS in OA.

## 2. Materials and Methods

### 2.1. Participant Recruitment and Criteria

Patients with osteoarthritis (OA) and healthy volunteers were recruited for this study. The study received ethical approval from the responsible regional ethics committees (ethics number 1332/2022, approval date: 12 May 2023 and EK GZ 13/2015-2018, approval date: 14 January 2013). Healthy volunteers were restricted to ≥50 years of age to minimize the risk of including individuals with subclinical or early-stage osteoarthritis, which becomes more prevalent beyond the fifth decade of life. Inclusion criteria for the OA group (*n* = 50) were as follows: (a) age over 40 years, (b) clinical and radiological diagnosis of knee osteoarthritis with a Kellgren–Lawrence grade of 2–4. Inclusion criteria for the healthy control group (*n* = 20) were as follows: (a) age over 50 years, (b) no known diagnosis of osteoarthritis, and (c) no current anticoagulant therapy. General exclusion criteria for all participants included the presence of systemic inflammatory diseases (e.g., rheumatoid arthritis), acute infections, malignancy, and intra-articular corticosteroid injections within the last three months.

### 2.2. ACS Preparation

ACS was prepared using a dedicated centrifugation system (IMPACT^®^, Plasmaconcept, Cologne, Germany), hereafter referred to as ICS (IMPACT Centrifuge System). A specialized test kit containing all necessary materials was used for ACS production. Whole blood (10 mL) was collected into single-use IMPACT ACS syringes (Plasmaconcept, Cologne, Germany) pre-filled with glass beads, which serve to activate leukocytes and platelets, thereby stimulating the production of a broad range of cytokines and growth factors. Following collection, the syringes were placed on a laboratory rotator at 10 rpm for 20 min to promote controlled coagulation. Subsequently, the syringe containing coagulated blood was connected to an empty satellite syringe via a connecting tube and placed into the ICS. Centrifugation was performed for 20 min at 940× *g* according to the proprietary ICS protocol. An integrated sensor system monitored the interface between the cellular and serum compartments and automatically controlled transfer of the conditioned serum into the satellite syringe. The resulting ACS was aliquoted and stored at −80 °C until further analysis.

### 2.3. Questionnaire

Demographic and lifestyle factors potentially influencing ACS composition were recorded via a standardized questionnaire. The assessed variables included smoking status (yes/no), alcohol consumption (daily/occasionally or rarely/never), body mass index (BMI; categorized as 18.5 < 25.0, 25.0 < 30.0, or >30.0 kg/m^2^), sex (male/female), and nutritional habits. Dietary patterns were categorized broadly into carnivorous or vegetarian/vegan to identify general trends, although specific nutrient intake was not recorded.

### 2.4. Multiplex Assay

Stored ACS aliquots were thawed and centrifuged to remove debris. Bio-Plex Pro assay (Bio-Rad Laboratories, Inc., Hercules, CA, USA) was performed according to the manufacturer’s instructions and evaluated using the Bio-Plex 200 Analyzer. In the assay, antibodies coupled to fluorescent magnetic beads allowed for the detection and quantification of each analyte. The following analytes were measured by multiplex assay, with their respective detection limits: pro- and anti-inflammatory cytokines—IL-1β (0.46 pg/mL), IL-1RA (14.55 pg/mL), IL-4 (0.21 pg/mL), IL-6 (0.24 pg/mL), IL-8 (0.51 pg/mL), IL-10 (0.72 pg/mL), IL-13 (0.18 pg/mL), IL-15 (24.2 pg/mL), IL-17a (2.6 pg/mL), IL-18 (0.92 pg/mL), and TNF-α (6.3 pg/mL); growth factors—VEGF-A (6.74 pg/mL), FGF (3.46 pg/mL), PDGF-BB (3.09 pg/mL), TGF-β1 (0.93 pg/mL), TGF-β2 (0.61 pg/mL), and TGF-β3 (0.58 pg/mL); and degradative enzymes—MMP2 (242.64 pg/mL) and MMP3 (377.06 pg/mL). Among platelet-derived growth factors, only PDGF-BB was quantified because it was the sole PDGF isoform included in the validated multiplex panel. While other PDGF isoforms (e.g., PDGF-AA, PDGF-AB) also play important roles in inflammation and tissue repair, they were not available in the current assay and therefore could not be assessed. Analytes below the detection limit (LOD) were assigned a value of LOD/2 for descriptive statistics and group comparison; handling for regression analyses is described in Section 2.6.

### 2.5. ELISA

Insulin-like Growth Factor 1 (IGF-1) levels in ACS were quantified using the Human IGF-1 ELISA Kit (Abcam, Cambridge, UK). Thawed and centrifuged aliquots were processed according to the manufacturer’s protocol. Absorbance was measured at 450 nm using the Synergy 2 Multiplate Reader (BioTek, Winooski, VT, USA) with Gen 5 software.

### 2.6. Statistical Analysis

All statistical analyses were performed using GraphPad Prism version 10 (GraphPad Software, San Diego, CA, USA). Mann–Whitney U tests were used for univariate comparisons between OA and healthy control groups. Subgroups defined by lifestyle variables, such as sex, alcohol consumption, smoking, diet, BMI, and age used a two-way ANOVA with Tukey’s multiple-comparison test, with the False Discovery Rate (FDR) method by Benjamini and Hochberg applied to account for multiple comparisons. To address demographic imbalances and potential confounding, multiple linear regression (MLR) analyses were performed for the primary outcome measures (MMP-2, MMP-3, and TGF-β isoforms). Biomarker concentrations were log_10_-transformed to satisfy normality assumptions, with group status (OA vs. healthy), age, and sex included as independent variables. Regression coefficients from log-transformed models were back-transformed to fold-changes using the following formula:

Fold-change = 10^β^.

Residual diagnostics, including Shapiro–Wilk tests for normality and variance inflation factors (VIF) for multicollinearity, were evaluated for all models. Furthermore, a post hoc power analysis was conducted to assess the robustness of comparisons given the unequal group sizes. Data in the text are presented as mean ± standard deviation (SD) to reflect the biological variability within each group, which is particularly relevant for understanding inter-individual differences in ACS composition. Data in boxplots display the median, interquartile range (IQR), and minimum and maximum values. Statistical significance was set at *p* < 0.05, and all tests were two-sided.

#### Multiple Linear Regression Modeling

For primary biomarkers (MMP-2, MMP-3, TGF-β1, TGF-β2, TGF-β3), multiple linear regression was performed with the following specifications:

(1) Data transformation: Biomarker concentrations were log_10_-transformed to address right-skewed distributions and stabilize variance. Transformation success was evaluated using Shapiro–Wilk tests for normality of residuals.
(2) Model specification: Each biomarker was modeled as: log_10_(Biomarker) ~ Group + Age + Sex + ε, where Group (OA = 1, Healthy = 0), Age (continuous, years), and Sex (Female = 1, Male = 0) were included as predictors.
(3) Model diagnostics: Residual normality was assessed using Shapiro–Wilk, Anderson–Darling, and Kolmogorov–Smirnov tests. Multicollinearity was evaluated using variance inflation factors (VIF), with VIF < 5 considered acceptable. Model fit was quantified using R^2^ and adjusted R^2^.
(4) Coefficient interpretation: Regression coefficients (β) from log-transformed models were back-transformed to fold-changes using 10^β^. A β-value of 0.3 corresponds to 10^0.3^ = 2.0-fold (100% increase); β = −0.1 corresponds to 10^−0.1^ = 0.79-fold (21% decrease).
(5) Missing data: Samples with biomarker values below detection limits were excluded from the respective regression model. This affected 5 samples for MMPs, 9 samples for TGF-β1 and TGF-β3, and 6 samples for TGF-β2.

## 3. Results

### 3.1. Comparison OA vs. Healthy—General Data

Fifty patients with osteoarthritis (OA; Kellgren–Lawrence grade 2–4) and twenty healthy volunteers over 50 were included in the study. The OA group comprised 19 males and 31 females, aged 41 to 85 (mean age: 64.35 years) and with body weights ranging from 56 to 135 kg (mean: 78.79 kg). The healthy group consisted of 4 males and 16 females, aged 50 to 63 years (mean: 55.25 years), with body weights ranging from 50 to 103 kg (mean: 65.35 kg). General characteristics showed that OA patients exhibit a higher mean body weight than healthy controls.

**Figure 1:**
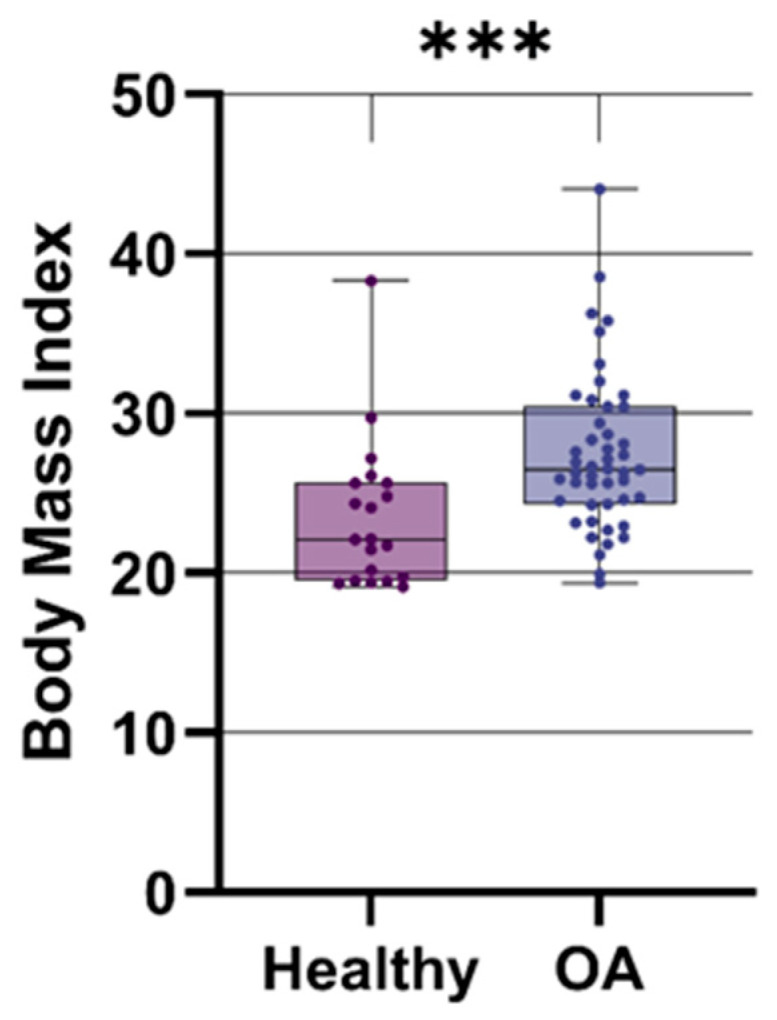
Body mass index (BMI) comparison between healthy individuals and OA patients. Data are presented as boxplots showing median, interquartile range (IQR), and minimum/maximum values. Statistical significance: *** *p* < 0.001 (Mann–Whitney U test with FDR correction).

As shown in Figure 1, analysis of demographic data revealed that OA patients exhibited a significantly higher Body Mass Index (BMI) compared to healthy controls. These baseline differences necessitated the use of adjusted statistical models for the subsequent molecular analyses.

### 3.2. Comparison OA vs. Healthy—Composition

**Figure 2:**
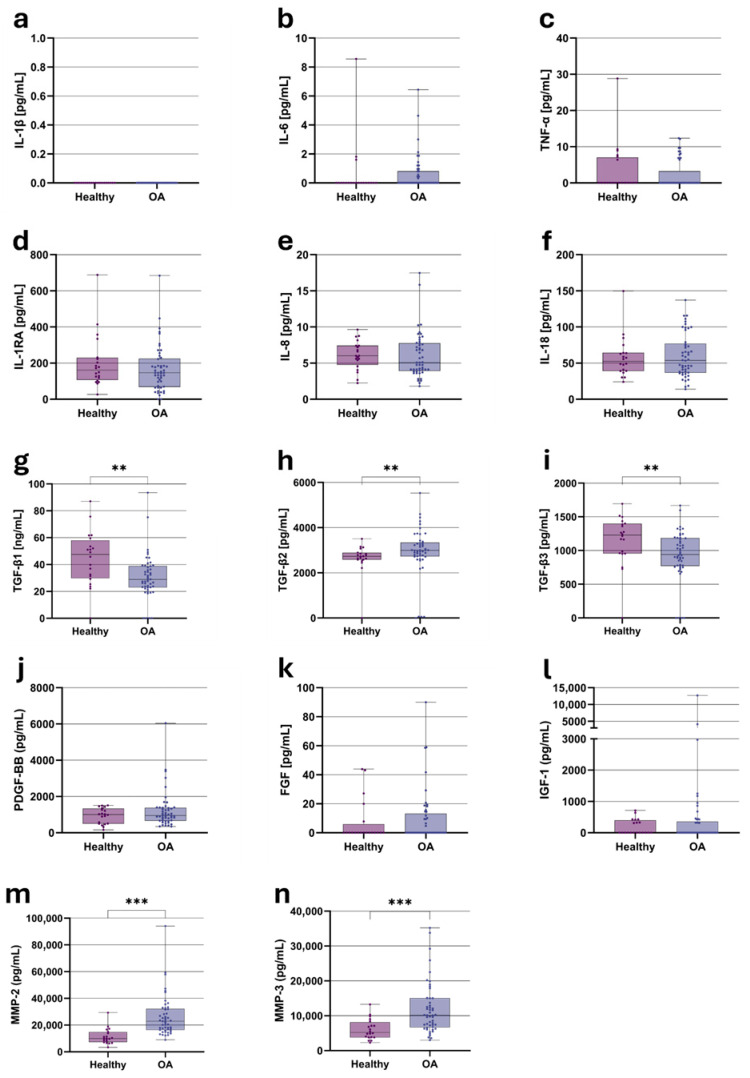
Concentrations of pro- and anti-inflammatory cytokines, growth factors, and matrix metalloproteinases in autologous conditioned serum (ACS) obtained from healthy individuals and osteoarthritis (OA) patients. (**a**) IL-1β; (**b**) IL-6; (**c**) TNF-α; (**d**) IL-1RA; (**e**) IL-8; (**f**) IL-18; (**g**) TGF-β1; (**h**) TGF-β2; (**i**) TGF-β3; (**j**) PDGF-BB; (**k**) FGF; (**l**) IGF-1; (**m**) MMP-2; (**n**) MMP-3. Cytokines, growth factors, and matrix metalloproteinases were quantified using a multiplex assay; IGF-1 concentrations were determined by ELISA. Data are presented as boxplots showing median, interquartile range (IQR), and minimum/maximum values. Statistical significance: ** *p* < 0.01, *** *p* < 0.001 (Mann–Whitney U test with FDR correction).

All ACS samples were analyzed for their pro- and anti-inflammatory cytokines, growth factors, and degradative enzymes. The cytokine analysis revealed that six of the 11 tested cytokines—IL-1β (Figure 2a), IL-4, IL-10, IL-13, IL-15, and IL-17a—were below the detection limit in all samples. Although IL-1β is a key proinflammatory cytokine implicated in OA pathogenesis, it remained undetectable. IL-6 and TNF-α, both associated with OA-related inflammation, were present at low levels, with no statistically significant differences between healthy and OA groups (Figure 2b,c). IL-1RA, an anti-inflammatory mediator, was consistently detectable across all groups (Figure 2d), exhibiting low variability. Median levels were slightly higher in healthy individuals (160.98 pg/mL) than in OA patients (146.32 pg/mL), though this difference did not reach statistical significance. This suggests that IL-1RA expression is not substantially modulated by OA status. Similarly, IL-8 and IL-18 (Figure 2e,f) showed stable concentrations across cohorts, with no significant differences. Median IL-8 levels were 6.00 pg/mL in healthy individuals and 5.08 pg/mL in OA patients, while IL-18 levels were 52.40 pg/mL and 53.74 pg/mL, respectively. These findings indicate limited alterations in these cytokines in OA and highlight their potential role as stable systemic markers rather than dynamic disease indicators.

Six out of seven growth factors analyzed were successfully detected, with VEGF-A being the only factor below the detection limit. Transforming growth factor beta (TGF-β), a key regulator of cellular differentiation, immune modulation, and tissue homeostasis, showed isoform-specific alterations between groups in univariate analyses. However, to address potential confounding by demographic differences, we performed age- and sex-adjusted multiple linear regression analyses for all TGF-β isoforms.

After log_10_-transformation to satisfy normality assumptions and adjusting for age and sex, TGF-β1 remained significantly lower in OA-derived ACS (0.77-fold, 95% CI: 0.62 to 0.95, *p* = 0.017), representing a 23% reduction compared to healthy controls. TGF-β3 showed a directionally consistent but non-significant trend toward lower levels in OA patients (0.89-fold, 95% CI: 0.78 to 1.03, *p* = 0.11). In contrast, TGF-β2 could not be reliably modeled using parametric regression due to severe violations of normality assumptions, even after log-transformation (Shapiro–Wilk W = 0.53, *p* < 0.0001). Non-parametric Mann–Whitney U test revealed no significant difference in TGF-β2 between groups (*p* = 0.89). Age was not a significant predictor for any TGF-β isoform (all *p* > 0.06), confirming that observed differences were attributable to OA status rather than age-related effects. Sex showed no significant association with TGF-β1 or TGF-β3 (both *p* > 0.08).

The adjusted regression models explained 17.9% (TGF-β1) and 12.7% (TGF-β3) of the variance in growth factor concentrations (adjusted R^2^). Multicollinearity diagnostics confirmed minimal correlation between predictors (all VIF < 1.3), supporting model validity. Residual analyses indicated that log-transformation successfully restored normality for TGF-β1 and TGF-β3 (Shapiro–Wilk *p* > 0.18), but not for TGF-β2, highlighting technical challenges in quantifying this particular isoform.

Platelet-derived growth factor BB (PDGF-BB, Figure 2j) was present at comparable levels in both groups, with 1003.74 pg/mL in healthy individuals and 947.14 pg/mL in OA patients. In contrast, fibroblast growth factor (FGF, Figure 2k) was only detectable in a subset of ACS samples, precluding the median value calculation. No significant differences were observed between the groups for this factor.

As illustrated in Figure 2l, Insulin-like Growth Factor 1 (IGF-1) was measured across all samples; however, in many cases, its concentration fell below the detection limit. As a result, no apparent differences between healthy individuals and OA patients were observed.

Matrix metalloproteinases MMP-2 and MMP-3—critical enzymes involved in extracellular matrix degradation—were detectable in both groups, with a marked trend toward higher concentrations in ACS obtained from OA patients (Figure 2m,n). To confirm these findings independently of demographic confounders, we performed age- and sex-adjusted multiple linear regression analyses with log_10_-transformed MMP concentrations.

After adjusting for age and sex, MMP-2 remained highly significantly elevated in OA-derived ACS (2.05-fold increase, 95% CI: 1.56 to 2.71, *p* < 0.001), corresponding to a 105% elevation compared to healthy controls. Similarly, MMP-3 was significantly elevated (1.64-fold increase, 95% CI: 1.23 to 2.18, *p* = 0.001), representing a 64% increase. Age was not a significant predictor for either MMP (both *p* > 0.25). Notably, sex emerged as a significant predictor specifically for MMP-3 (*p* < 0.001), with male participants exhibiting 1.75-fold higher MMP-3 concentrations than females, independent of OA status and age. This sex-specific regulation was not observed for MMP-2 (*p* = 0.47).

The adjusted models for MMPs explained substantial variance (adjusted R^2^: 38.9% for MMP-2; 37.1% for MMP-3), and all normality tests were satisfied after log-transformation (Shapiro–Wilk *p* > 0.29). VIF values remained below 1.3, confirming absence of multicollinearity. These findings demonstrate that the elevation in degradative enzymes in OA-derived ACS is robust and independent of demographic variables.

### 3.3. Comparison OA vs. Healthy—Exploratory Questionnaire Analysis

The questionnaire evaluation, as described in the Section 2, aimed to determine whether specific demographic and clinical parameters influence the composition of ACS. However, given that the sample size in certain subgroups (e.g., smokers, specific dietary groups) was limited, the following analyses are considered strictly exploratory and hypothesis-generating. Consequently, these findings serve primarily to identify potential trends that may guide future therapeutic stratification, rather than providing definitive evidence. Therefore, only statistically significant results or clear trends are reported for each subgroup.

#### 3.3.1. Sex

**Figure 3:**
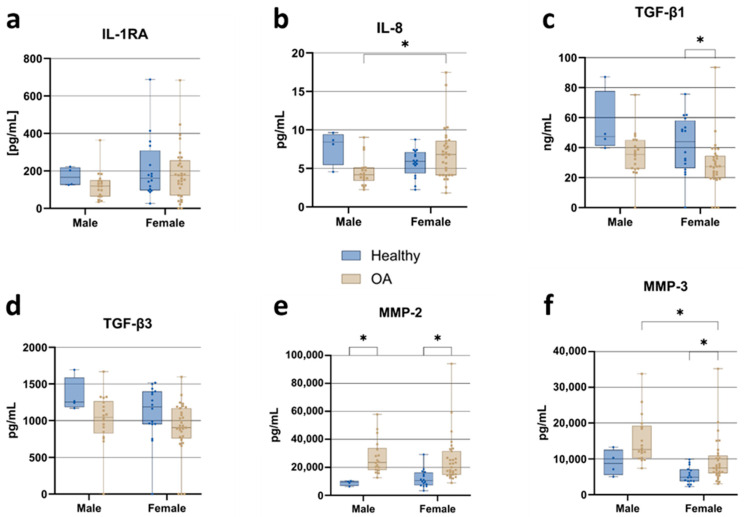
Sex-specific stratification of cytokines, TGF-β isoforms, and matrix metalloproteinases in ACS samples from healthy individuals and OA patients. (**a**) IL-1RA; (**b**) IL-8; (**c**) TGF-β1; (**d**) TGF-β3; (**e**) MMP-2; (**f**) MMP-3. Data are presented as boxplots showing median, interquartile range (IQR), and minimum/maximum values. Statistical significance: * *p* < 0.05 (two-way ANOVA with Tukey’s post hoc test and FDR correction).

Sex-specific differences in cytokine and growth factor profiles were identified in healthy individuals and OA patients. These exploratory stratified analyses complement the primary adjusted regression models (Table 1), which identified a significant sex-specific effect for MMP-3 but not for other biomarkers. IL-1RA levels (Figure 3a) showed a trend toward higher levels in healthy men than in male OA patients, a pattern not observed in women. IL-8 concentrations (Figure 3b) showed a significant sex-dependent variation within the OA cohort, where male OA patients exhibited significantly lower IL-8 levels than their female counterparts. In the case of TGF-β isoforms, TGF-β1 levels (Figure 3c) were significantly reduced in female OA patients compared to healthy female controls. At the same time, the male groups showed a similar, non-significant trend. TGF-β3 levels (Figure 3d) showed a similar, non-significant trend in both sexes. The analysis of matrix metalloproteinase (MMPs; Figure 3e,f) revealed elevated MMP-2 and MMP-3 levels in OA patients of both sexes. MMP-2 levels were significantly higher in male and female OA patients than in their healthy counterparts. Similarly, MMP-3 concentrations were significantly elevated in female OA patients compared to their healthy counterparts, while a non-significant trend was observed in males. MMP-3 levels were also significantly higher in male OA patients compared to female OA patients, consistent with the sex-specific effect identified in the adjusted regression analysis (Table 1), where males showed 1.75-fold higher MMP-3 concentrations independent of OA status.

#### 3.3.2. Alcohol

**Figure 4:**
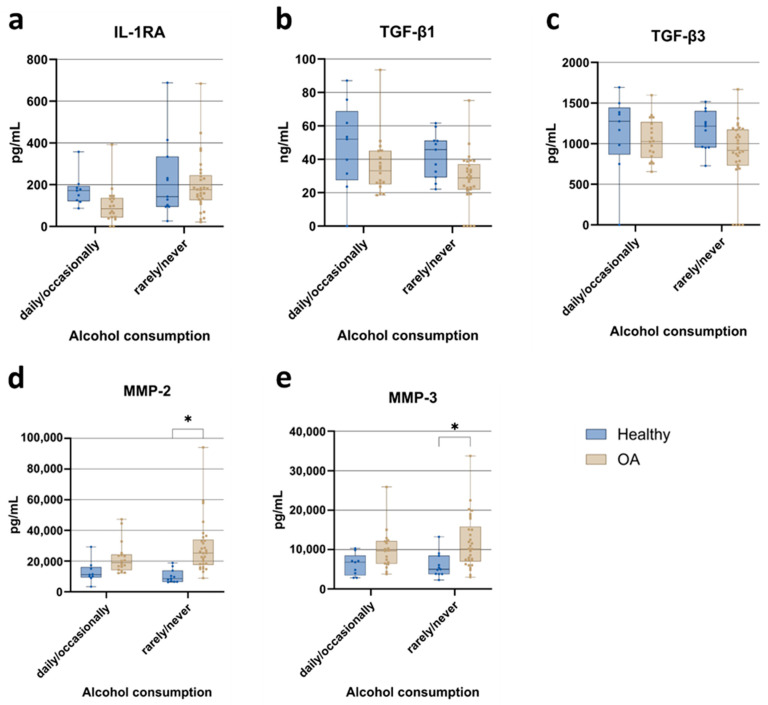
Comparison of cytokine, TGF-β isoform, and matrix metalloproteinase concentrations in autologous conditioned serum (ACS) samples from healthy individuals and osteoarthritis (OA) patients, stratified by alcohol consumption. (**a**) IL-1RA; (**b**) TGF-β1; (**c**) TGF-β3; (**d**) MMP-2; (**e**) MMP-3. Data are presented as boxplots showing median, interquartile range (IQR), and minimum/maximum values. Statistical significance: * *p* < 0.05 (two-way ANOVA with Tukey’s post hoc test and FDR correction).

Due to the limited sample size in specific subgroups, individuals reporting daily and occasional alcohol consumption were pooled into a single category, while those consuming alcohol rarely or never were merged into another. Overall, alcohol consumption frequency did not exert a statistically significant effect on cytokine concentrations in ACS when comparing healthy individuals and OA patients. Nevertheless, IL-1RA levels (Figure 4a) showed a trend toward lower concentrations in OA patients with more frequent alcohol intake compared to their healthy controls. Similarly, TGF-β1 and TGF-β3 isoforms (Figure 4b,c) did not differ significantly between groups; however, a consistent trend toward lower levels was observed in OA patients irrespective of alcohol consumption. Matrix metalloproteinase (MMP) levels (Figure 4d,e) were also not influenced by alcohol intake frequency. Still, the expected increase in MMP concentrations in OA patients was confirmed, showing significantly elevated levels in ACS compared to healthy controls, specifically within the group that consumed alcohol rarely or never.

#### 3.3.3. Smoking

**Figure 5:**
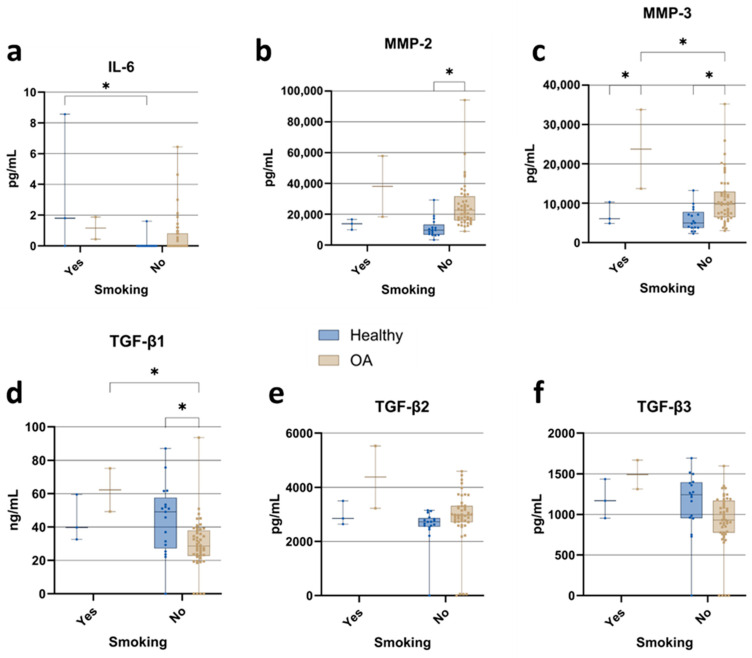
Impact of smoking status on ACS composition: comparison of cytokine, matrix metalloproteinase, and TGF-β isoform concentrations in samples from healthy individuals and osteoarthritis (OA) patients. (**a**) IL-6; (**b**) MMP-2; (**c**) MMP-3; (**d**) TGF-β1; (**e**) TGF-β2; (**f**) TGF-β3. Data are presented as boxplots showing median, interquartile range (IQR), and minimum/maximum values. Statistical significance: * *p* < 0.05 (two-way ANOVA with Tukey’s post hoc test and FDR correction).

Smoking status was recorded to explore its potential influence on cytokine, MMP, and growth factor levels in ACS from healthy individuals and OA patients (Figure 5). However, as the number of smokers, particularly within the OA cohort, was small, the subgroup analyses must be interpreted cautiously and considered exploratory. Within the healthy cohort, IL-6 concentrations were higher in smokers than in non-smokers (Figure 5a), whereas no significant IL-6 differences were detected between smoking and non-smoking OA patients. MMP-2 levels were higher in OA patients than in healthy individuals among non-smokers, with a similar, but non-significant, pattern observed in smokers (Figure 5b). MMP-3 concentrations were increased in OA patients compared with healthy individuals, irrespective of smoking status (Figure 5c). Among the TGF-β isoforms, only TGF-β1 showed a significant association with smoking status, whereas TGF-β2 and TGF-β3 were not markedly affected. TGF-β1 concentrations (Figure 5d) were significantly lower in non-smoking OA patients than in both smoking OA patients and non-smoking healthy individuals. In contrast, TGF-β2 (Figure 5e) and TGF-β3 (Figure 5f) did not differ significantly by smoking status in either healthy participants or OA patients, although some numerical differences between subgroups were observed.

#### 3.3.4. Nutrition

**Figure 6:**
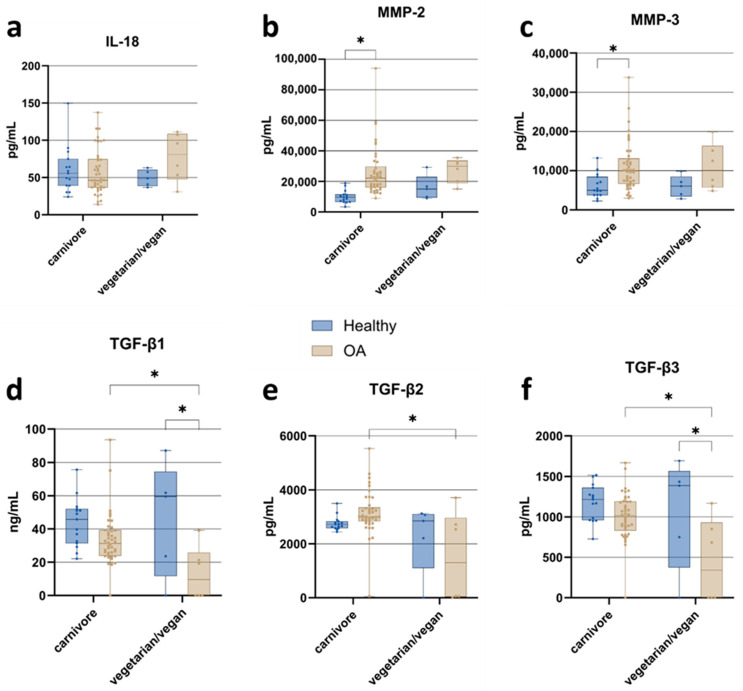
Impact of dietary habits on ACS composition: comparison of cytokine, matrix metalloproteinase, and TGF-β isoform concentrations in samples from healthy individuals and osteoarthritis (OA) patients, stratified by carnivore versus vegetarian/vegan diet. (**a**) IL-18; (**b**) MMP-2; (**c**) MMP-3; (**d**) TGF-β1; (**e**) TGF-β2; (**f**) TGF-β3. Data are presented as boxplots showing median, interquartile range (IQR), and minimum/maximum values. Statistical significance: * *p* < 0.05 (two-way ANOVA with Tukey’s post hoc test and FDR correction).

Cytokine and growth factor concentrations were quantified in healthy and OA cohorts stratified by dietary habits (carnivorous vs. vegetarian/vegan) to evaluate potential dietary influences on inflammation and tissue remodeling pathways (Figure 6). IL-18 levels (Figure 6a) were markedly elevated in vegetarian/vegan OA patients compared to carnivorous OA patients and healthy individuals of both dietary groups. MMP-2 concentrations (Figure 6b) were significantly elevated in carnivorous OA patients compared to carnivorous healthy individuals. A similar trend was observed within the vegetarian/vegan group, with OA patients exhibiting higher MMP-2 levels than their healthy counterparts. MMP-3 levels (Figure 6c) were also significantly elevated in carnivorous OA patients compared to healthy carnivores and were consistently higher in OA patients overall compared to healthy individuals, independent of dietary group. Variations in TGF-β isoforms further reflect dietary influences: TGF-β1 (Figure 6d), -β2 (Figure 6e), and -β3 (Figure 6f) were all significantly higher in carnivorous OA patients compared to their vegetarian/vegan counterparts. Additionally, TGF-β1 and TGF-β3 levels were significantly higher in healthy vegetarian/vegan individuals than in vegetarian/vegan OA patients.

#### 3.3.5. Body Mass Index (BMI)

**Figure 7:**
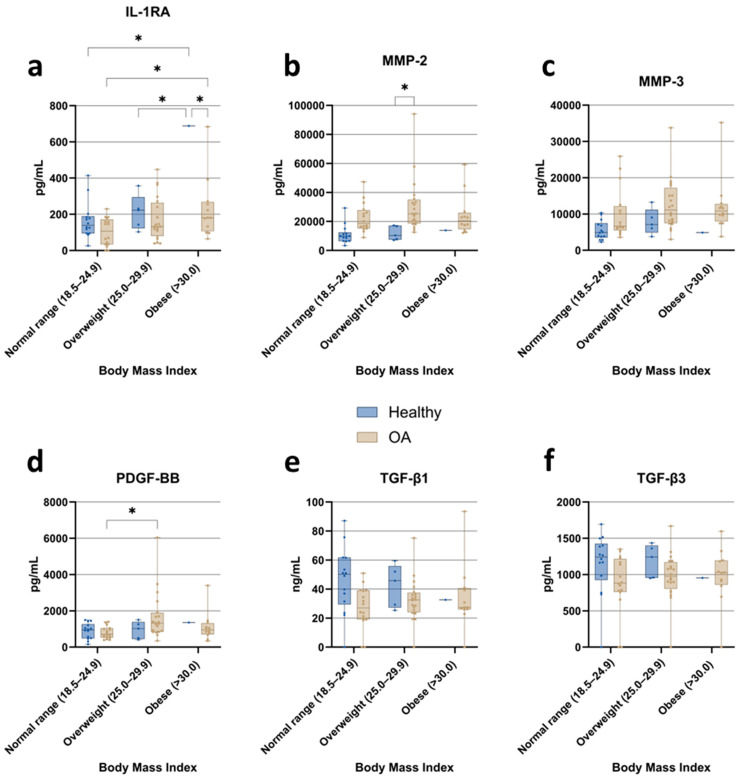
Impact of body mass index (BMI) on ACS composition: comparison of cytokine, matrix metalloproteinase, growth factor, and TGF-β isoform concentrations in samples from healthy individuals and osteoarthritis (OA) patients, stratified by BMI category (normal range, overweight, obese). (**a**) IL-1RA; (**b**) MMP-2; (**c**) MMP-3; (**d**) PDGF-BB; (**e**) TGF-β1; (**f**) TGF-β3. Data are presented as boxplots showing median, interquartile range (IQR), and minimum/maximum values. Statistical significance: * *p* < 0.05 (two-way ANOVA with Tukey’s post hoc test and FDR correction).

For the analysis, body mass index (BMI) was stratified into three categories: normal range, overweight, and obese. Cytokine levels were then compared between healthy individuals and OA patients across these BMI groups. Among the cytokines assessed, only IL-1RA (Figure 7a) showed a significant difference between BMI categories, with higher concentrations in overweight than in normal-weight individuals in both the healthy and OA cohorts. No relevant BMI-associated changes were observed for the remaining cytokines. Interpretation of data in the obese healthy group was constrained by the presence of only one participant, which markedly limits statistical power in this category. Matrix metalloproteinase (MMP) concentrations (Figure 7b,c) were substantially higher in OA patients than in healthy individuals across BMI categories. MMP-2 levels were significantly elevated in overweight OA patients compared with overweight healthy controls. When comparing BMI categories within the OA group, MMP-2 and MMP-3 did not show a consistent stepwise increase from normal weight to overweight to obesity; concentrations in obese OA patients were comparable to, or slightly lower than, those observed in overweight OA patients. Regarding growth factors, PDGF-BB levels (Figure 7d) were significantly higher in overweight OA patients than in OA patients with normal BMI. In contrast, levels in obese OA patients were similar to those in the normal-weight group, again indicating a non-linear relationship between BMI and PDGF-BB. Members of the TGF-β family, TGF-β1 (Figure 7e) and TGF-β3 (Figure 7f), remained relatively stable across BMI categories but were consistently lower in OA patients than in healthy individuals.

#### 3.3.6. Age

**Figure 8:**
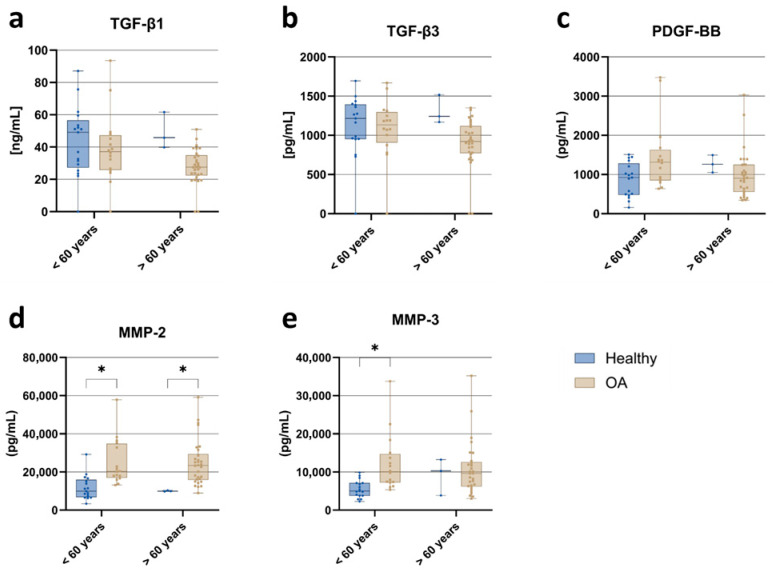
Age-related differences in ACS composition: comparison of growth factor and matrix metalloproteinase concentrations in samples from healthy individuals and osteoarthritis (OA) patients, stratified by age group (<60 years vs. >60 years). (**a**) TGF-β1; (**b**) TGF-β3; (**c**) PDGF-BB; (**d**) MMP-2; (**e**) MMP-3. Data are presented as boxplots showing median, interquartile range (IQR), and minimum/maximum values. Statistical significance: * *p* < 0.05 (two-way ANOVA with Tukey’s post hoc test and FDR correction).

To investigate the influence of age on ACS composition, participants were stratified into two age groups: under 60 and 60 years or older, including both healthy individuals and OA patients. A trend toward higher concentrations of TGF-β1 and TGF-β3 (Figure 8a,b) was observed in individuals under 60, with the difference more pronounced among OA patients than healthy controls. OA patients exhibited lower levels of TGF-β1 and TGF-β3 than healthy individuals, regardless of age. In contrast, PDGF-BB levels (Figure 8c) were not significantly different between groups. Matrix metalloproteinases were significantly elevated in OA patients compared to healthy individuals. MMP-2 (Figure 8d) was higher in both age groups, whereas MMP-3 (Figure 8e) reached significance only in the under-60 group.

## 4. Discussion

Autologous conditioned serum (ACS) represents a promising therapeutic approach for osteoarthritis (OA), leveraging endogenous biological mechanisms to modulate inflammation and facilitate repair. In the present study, we sought to address remaining knowledge gaps by comprehensively characterizing the cytokine, growth factor, and degradative enzyme profiles of ACS derived from OA patients and healthy individuals and by evaluating how demographic and lifestyle factors modulate these profiles. Three main observations emerged: first, ACS generated with a short-incubation protocol contained very low levels of classical pro-inflammatory cytokines, while IL-1RA was consistently present in both OA patients and healthy donors; second, ACS from OA patients displayed a catabolic shift compared with healthy donors, with higher MMP-2/MMP-3 and lower TGF-β1/TGF-β3 concentrations; and third, sex, BMI, smoking status, diet, alcohol consumption, and age each contributed to substantial inter-individual variability in ACS composition. Together, these findings provide a biological basis for moving toward a more stratified application of ACS in OA.

ACS has demonstrated mild to moderate effectiveness in alleviating OA symptoms, with clinical benefits persisting for up to two years post-treatment [11,17], but several critical aspects of ACS therapy remain insufficiently understood, including precise mechanisms of action, long-term efficacy, and patient-specific response variability [18]. A key molecular finding of the present study was the absence of detectable proinflammatory IL-1β and very low levels of IL-6 and TNF-α in ACS samples from both OA patients and healthy individuals. Given their established roles in cartilage degradation and synovial inflammation [5,19], their minimal presence supports the therapeutic goals of ACS. In contrast, IL-1 receptor antagonist (IL-1RA) levels were consistently high in both cohorts, which is consistent with previous findings indicating that IL-1RA can be robustly induced by the ACS preparation protocol irrespective of disease state [20].

A critical consideration is the concentration of the anti-inflammatory cytokine IL-1RA. The levels of approximately 200 pg/mL observed in our study are lower than those reported in studies utilizing 24-h incubation protocols, which can exceed 1000 pg/mL [11]. Whether these concentrations are biologically sufficient for a therapeutic effect remains uncertain. Previous studies have suggested that IL-1RA concentrations exceeding 500–1000 pg/mL may be required to effectively compete with IL-1β for receptor binding in the joint microenvironment [4,11]. Thus, the IL-1RA levels achieved with the short-incubation IMPACT protocol may represent a biologically distinct product compared to conventional, long-incubation ACS preparations. While longer incubation durations—up to 24 h—can significantly increase IL-1RA levels [7,20], these extended times also lead to a concomitant rise in proinflammatory cytokines, which may counteract the desired anti-inflammatory effects [9,15,21]. Consequently, our data suggest that the short incubation produces a “cleaner” anti-inflammatory profile—albeit with lower absolute IL-1RA concentrations—by virtually eliminating IL-1β and TNF-α. This trade-off raises an important clinical question: Is a lower IL-1RA concentration with minimal pro-inflammatory contamination therapeutically superior to higher IL-1RA levels accompanied by elevated IL-1β and TNF-α? The answer likely depends on the net balance of pro- and anti-inflammatory signals within the injected product, which cannot be determined from compositional data alone [4]. Previous studies have established that a 10- to 100-fold molar excess of IL-1RA is required to achieve meaningful inhibition of IL-1-mediated signaling, suggesting that the ratio of mediators may be more clinically relevant than absolute concentrations alone [22,23]. Future clinical trials directly comparing short- versus long-incubation ACS protocols are needed to determine whether this specific cytokine balance results in equivalent or superior clinical efficacy.

A major methodological advancement of the present study was the application of age- and sex-adjusted multiple linear regression analyses to account for demographic imbalances between groups (OA: *n* = 50, mean age 64.4 years, 62% female; Healthy: *n* = 20, mean age 55.3 years, 80% female). This analytical approach directly addresses potential confounding and strengthens the validity of our findings by demonstrating that observed biomarker differences are attributable to OA status rather than demographic factors.

ACS also contains growth factors relevant to tissue regeneration, including TGF-β1 to TGF-β3, PDGF-BB, FGF, and IGF-1. Our analyses revealed that TGF-β1 concentrations remained significantly lower in OA-derived ACS even after controlling for age and sex (0.77-fold, *p* = 0.017), underscoring a disease-specific reduction in this cartilage-maintaining growth factor. TGF-β3 showed a consistent directional trend (0.89-fold, *p* = 0.110), though not reaching statistical significance. Importantly, age was not a significant predictor for either isoform (*p* > 0.060), confirming that these differences reflect OA pathophysiology rather than age-related decline. The opposing pattern of higher TGF-β2 but lower TGF-β1 and TGF-β3 in OA-derived ACS in univariate analyses is compatible with isoform-specific regulation of TGF-β signaling in the OA microenvironment, with a relative loss of the cartilage-maintaining isoforms TGF-β1 and TGF-β3 and a concomitant shift towards TGF-β2, in line with isoform-specific roles of TGF-β signaling in cartilage homeostasis and OA [24,25]. However, the technical challenges encountered in modeling TGF-β2 parametrically—due to high inter-individual variability and a substantial proportion of values near the detection limit—preclude definitive conclusions regarding this particular isoform. This highlights the need for more sensitive analytical methods or alternative quantification approaches for TGF-β2 in future studies.

PDGF-BB and FGF levels appeared unaffected by disease status, suggesting their stable expression. However, their presence remains beneficial due to their capacity to stimulate cellular proliferation, angiogenesis, and tissue repair mechanisms [26,27]. IGF-1 frequently remained below the detection threshold, likely due to the short incubation period, whereas prior studies reported higher levels after extended incubation times [10,21]. Therefore, future efforts should optimize ACS preparation protocols—specifically incubation times—to enhance IGF-1 concentrations, given its pivotal role in promoting proteoglycan synthesis and chondrocyte viability [28] and in achieving an ideal therapeutic cytokine profile.

Based on the regression analyses, our data show significantly elevated levels of matrix metalloproteinases (MMP-2: 2.05-fold, *p* < 0.001; MMP-3: 1.64-fold, *p* = 0.001) in ACS from OA patients, independent of age and sex, reflecting enhanced extracellular matrix turnover, a hallmark of the disease [4,25]. The robustness of these findings is supported by high adjusted R^2^ values (38.9% and 37.1%, respectively) and the absence of confounding by demographic variables (age *p* > 0.25 for both MMPs).

A novel finding of this study is the identification of sex-specific regulation of MMP-3, with males exhibiting 1.75-fold higher concentrations than females (*p* < 0.001), independent of OA status and age. This sex-specific effect was not observed for MMP-2 (*p* = 0.47), suggesting differential hormonal or genetic regulation of these two metalloproteinases. MMP-3 (stromelysin-1) expression has been shown to be modulated by sex hormones in various tissues, potentially explaining the observed sex dimorphism [29]. In contrast, MMP-2 (gelatinase A) appears less susceptible to sex hormone modulation. This finding has important implications for personalized medicine approaches, as male OA patients may have intrinsically higher baseline MMP-3 activity, potentially influencing disease progression and treatment response. Future studies should investigate whether sex-stratified therapeutic strategies or sex-specific biomarker thresholds improve clinical outcomes in ACS therapy.

The interpretation of these elevated MMPs in a therapeutic product is complex. Although MMPs are known to be involved in tissue remodeling and removal of degradation products—processes necessary for repair [30]—their significantly higher concentration in ACS from OA suggests a persistent catabolic footprint. Unlike anti-inflammatory cytokines, this “disease trait” is not reversed by the manufacturing process. The analyses confirm that this catabolic signature is inherent to OA status and cannot be attributed to age or sex differences between cohorts. Therefore, it remains to be clarified whether the net effect of injecting these MMPs is neutralized by the simultaneously present anti-inflammatory mediators or whether they represent a potential catabolic risk that may exacerbate the OA environment. The concentrations are generally considered to exacerbate the OA environment by accelerating extracellular matrix breakdown and perpetuating inflammation. These findings are consistent with previous reports demonstrating that increased MMP activity is associated with structural progression and symptom burden in OA, thereby reinforcing the concept that MMPs represent key mediators of the catabolic joint environment [25,31]. Although high MMP levels could be perceived as detrimental, the pronounced anti-inflammatory environment within ACS may mitigate these activities. This hypothesis, however, warrants further experimental validation. Therefore, increased MMP levels might contribute positively by aiding the clearance of damaged matrix, facilitating subsequent regenerative processes.

A notable finding of the present study is the observed inter-individual variability in cytokine and growth factor levels, even within demographically similar groups. This variability is likely multifactorial. Beyond the lifestyle factors analyzed, it may be influenced by genetic predispositions, co-morbidities not captured in our questionnaire, medication history, and the specific location and severity of osteoarthritis within the included Kellgren–Lawrence grades 2–4. This underscores the complexity of the systemic biological environment and highlights the challenge in predicting the composition of autologous biologics. These observations mirror similar findings in platelet-rich plasma research, where variability in cytokine and growth factor content is a major determinant of clinical response, and emphasize the need for better characterization of patient-related sources of heterogeneity [7,32].

While the ACS preparation protocol used in this study was standardized, variations in incubation time, freeze–thaw cycles, and analytical methods may influence the stability and bioactivity of ACS components. Compared to PRP, ACS avoids exogenous additives (e.g., EDTA, citrate), more closely reflecting physiological conditions. Still, the observed heterogeneity across ACS studies underscores the need for methodological standardization to ensure consistent therapeutic outcomes. Addressing these methodological inconsistencies is essential to maximizing therapeutic effectiveness and ensuring reproducibility across clinical applications. This challenge similarly impacts PRP, where variability in cytokine and growth factor concentrations also significantly affects clinical outcomes. Standardized reporting of preparation protocols, incubation conditions, and analytical methods will therefore be crucial to enable meaningful comparisons between studies and to develop evidence-based recommendations for ACS use [9,32].

A novel aspect of this study was the use of a questionnaire to explore how demographic and lifestyle factors may influence cytokine and growth factor profiles in ACS. However, given that the sample size in certain subgroups (e.g., smokers, specific dietary groups) was limited, the following analyses are considered strictly exploratory and hypothesis-generating.

Sex-specific differences were evident: male OA patients exhibited lower IL-1RA levels and higher IL-8 and MMP-3 levels than females. These observations align with previous research, suggesting that hormonal influences significantly modulate inflammatory profiles [33]. For instance, testosterone has been linked to increased production of proinflammatory cytokines such as IL-6 and TNF-α. In contrast, estrogen promotes anti-inflammatory cytokines such as IL-1RA and IL-10, highlighting sex-specific cytokine modulation as a critical consideration in ACS therapy [33,34]. Although causality cannot be inferred from the present data, they support the concept that sex-specific biology may contribute to differential ACS composition and should be considered in future stratified analyses and clinical trial designs.

Lifestyle factors, such as alcohol consumption, also appeared to impact IL-1RA levels, with numerically lower concentrations among patients with higher alcohol intake, although these differences did not reach statistical significance. Chronic alcohol exposure is known to exacerbate inflammatory states through various mechanisms [35]. Here, oxidative stress, altered gut permeability, and systemic cytokine dysregulation, which are potentially influencing the therapeutic efficacy of ACS treatment, were reported in previous studies [36,37,38]. Furthermore, alcohol can disrupt immune function by altering cytokine production and impairing leukocyte function, thus possibly attenuating the beneficial anti-inflammatory effects of ACS therapies in OA patients who consume alcohol frequently. Due to the modest subgroup sizes, these findings should be regarded as exploratory.

Similarly, smoking was associated with significant changes. Healthy smokers showed elevated pro-inflammatory IL-6 levels, and within the OA cohort, smokers exhibited significantly higher levels of the degradative enzyme MMP-3 and the growth factor TGF-β1 compared to non-smokers, echoing findings from a pulmonary cell study [39]. Given the established relationship between smoking, systemic inflammation, endothelial dysfunction, and accelerated tissue damage [40], these observations support the notion that smoking may unfavorably influence the molecular profile of ACS. Cigarette smoking exposure triggers inflammatory responses through oxidative stress and activation of nuclear factor-kappa B (NF-κB) signaling pathways, subsequently elevating proinflammatory cytokines and MMPs, which can accelerate cartilage breakdown [41,42]. Thus, clinicians should prioritize advising OA patients regarding smoking, which can be an integral component of managing their disease progression and improving therapeutic outcomes. However, smoking-related subgroup analyses were based on very small sample sizes and should therefore be regarded as descriptive. These patterns require confirmation in larger, adequately powered cohorts.

Dietary impacts yielded intriguing insights: OA patients following vegetarian or vegan diets tended to exhibit elevated IL-18 levels compared with their carnivorous counterparts. This observation contrasts with the widespread perception of plant-based diets as inherently anti-inflammatory, underscoring the complex interplay between nutrition and inflammatory pathways. Importantly, even vegetarian diets may include specific animal-derived components, and existing evidence suggests that the reduction—rather than total exclusion—of red meat and dairy products plays a more pivotal role in modulating inflammatory markers [43]. MMP levels, in contrast, appeared largely unaffected by dietary habits, reinforcing their strong association with OA pathology irrespective of nutritional patterns [44,45]. Variations in TGF-β isoforms further reflect dietary influences: TGF-β1, -β2, and -β3 were all significantly higher in carnivorous OA patients compared to vegetarian/vegan individuals. These elevations may indicate diet-dependent effects on anabolic signaling cascades, given the essential role of TGF-β in cartilage repair and homeostasis [46]. These findings collectively emphasize the nuanced relationship between dietary patterns and OA’s inflammatory and anabolic molecular milieu. Nevertheless, the dietary subgroup sizes were limited, and potential confounding by other lifestyle factors cannot be excluded. A more granular understanding of specific dietary constituents and their mechanistic roles may aid in optimizing dietary strategies for disease modulation [43,47].

Consistent with the established link between obesity and inflammation, our BMI analysis revealed that OA patients with elevated BMI had higher MMP-2 levels. This finding supports a previous study that detected elevated MMP levels in synovial fluid, thereby linking adiposity to inflammatory processes in OA [48]. Excessive adipose tissue, particularly visceral fat, secretes proinflammatory adipokines such as leptin [49], resistin, and cytokines including IL-6 or TNF-α, all of which contribute to chronic low-grade inflammation and cartilage degradation in OA [50,51,52]. Furthermore, IL-1RA levels were significantly higher in overweight individuals compared to those with normal BMI in both cohorts, suggesting a compensatory upregulation of anti-inflammatory mechanisms with increasing adiposity. This could indicate that ACS derived from overweight donors may contain a more pronounced IL-1RA signal, potentially counterbalancing the proinflammatory milieu associated with excess adipose tissue. Whether this translates into superior clinical efficacy of ACS in overweight compared with normal-weight patients, however, remains to be tested in prospective outcome studies. In contrast, the growth factor PDGF-BB was significantly elevated only in overweight OA patients compared to normal-weight patients, whereas values in obese OA patients were similar to those in the normal-weight group, indicating a non-linear relationship between BMI and PDGF-BB. Our findings regarding BMI-related changes in PDGF-BB differ from those reported by O’Shaughnessey et al. for autologous protein solution (APS), who observed an enhanced profile of anabolic growth factors in obese patients [53]. These discrepancies may reflect fundamental differences between ACS and APS manufacturing (e.g., use of cell concentrates versus serum, distinct activation protocols) as well as differences in patient populations and analytical platforms. Thus, BMI-related effects on orthobiologic products appear to be product- and protocol-specific rather than generalizable across preparations. Because the healthy obese subgroup comprised only a single participant, no meaningful conclusions can be drawn regarding ACS composition in obese healthy individuals, and any apparent differences must be interpreted with extreme caution. Together with the well-established systemic effects of obesity on inflammation, this highlights the complex and multifactorial relationship between obesity and OA and underscores the need for therapeutic strategies that go beyond simple weight reduction.

Our age-stratified analysis revealed that younger OA patients (<60 years) showed numerically higher levels of regenerative growth factors, such as PDGF-BB and TGF-β isoforms. Disease status (OA vs. Healthy) was a much stronger determinant of MMP levels than age, as MMP-2 and MMP-3 were significantly elevated in OA patients across age groups. The enhanced regenerative potential coexisting with heightened disease activity in younger individuals suggests a critical therapeutic window for early ACS interventions, particularly given that the underlying disease process—rather than age alone—is the dominant driver of the degradative enzyme profile [54]. In contrast, healthy individuals did not exhibit such age-related variations, underscoring the disease-specific impact of OA on ACS composition [55]. The regenerative activity in younger patients may thus offer improved opportunities for ACS-based therapies to delay disease progression and promote cartilage repair [24,54]. Early diagnosis and timely intervention could optimize clinical outcomes in this patient subgroup.

### 4.1. Limitations

Despite the strengths of this study, several limitations should be acknowledged. First, there is an imbalance between the study groups (OA *n* = 50 vs. Healthy *n* = 20) and demographic imbalances in age and sex distribution. We addressed this issue by performing age- and sex-adjusted multiple linear regression analyses for key biomarkers, which confirmed the robustness of our main findings; nevertheless, residual confounding cannot be completely ruled out. Second, no a priori power analysis was performed, and sample sizes for lifestyle subgroups (e.g., smokers, vegans) were small. Post hoc power analysis using G*Power (version 3.1.9.7) (linear multiple regression, medium effect size *f^2^* = 0.15, α = 0.05, 3 predictors, *n* = 65) indicated 80% power to detect medium effect sizes in our primary regression models, confirming adequate sample size for main group comparisons. However, stratified subgroup analyses remain underpowered and should be interpreted as exploratory and hypothesis-generating.

Third, the dietary classification used (carnivore vs. vegetarian/vegan) is very general and does not take into account the consumption of processed foods or the intake of specific nutrients, which limits mechanistic conclusions. Fourth, the coagulation process, central to ACS preparation, could potentially lead to trapping and loss of specific bioactive molecules in the fibrin clot; this aspect was not investigated. Fifth, serum samples were not collected for a direct baseline comparison, and all ACS samples were frozen prior to analysis, which could have affected analyte stability [56]. Sixth, the technical challenges in quantifying TGF-β2—including higher inter-individual variability, substantial proportions below detection limits, and failure to achieve normality despite log-transformation—highlight the need for more sensitive or alternative analytical approaches for this particular cytokine in future ACS studies. The use of log-transformation and rigorous residual diagnostics strengthened the validity of parametric modeling, though TGF-β2 remained refractory to normalization, necessitating non-parametric analysis for this specific analyte. Furthermore, only PDGF-BB was measured, which does not capture the full spectrum of platelet-derived growth factor isoforms potentially relevant for ACS biology.

### 4.2. Clinical Relevance

From a clinical perspective, the present study underscores that ACS is not a ‘one-size-fits-all’ therapy. The data suggest that clinicians should consider patient-specific factors such as BMI, smoking status, and age when discussing the potential efficacy and prognosis of ACS treatment. Moreover, the regression analyses demonstrate that the molecular composition of ACS is fundamentally altered in OA patients, characterized by a catabolic shift (2-fold elevation in MMPs) concurrent with reduced anabolic capacity (23% reduction in TGF-β1). Importantly, these disease-related signatures persist after controlling for age and sex, indicating that OA pathophysiology—rather than demographic factors—is the primary driver of ACS heterogeneity.

The identification of sex-specific MMP-3 regulation (males 1.75-fold higher) suggests that future clinical trials should consider sex-stratified analyses to determine whether treatment response differs between male and female OA patients. Additionally, the technical difficulties encountered with TGF-β2 quantification underscore the importance of validating analytical methods and establishing clear detection thresholds before incorporating specific biomarkers into clinical decision-making algorithms. For example, the numerically lower regenerative growth factors in older patients might suggest that earlier intervention could be more beneficial. These insights pave the way for a more stratified approach to ACS therapy, potentially improving patient selection and optimizing treatment outcomes. The robust demonstration that MMPs and TGF-β alterations are independent of age suggests that disease stage or severity—rather than patient age per se—should be the primary consideration when selecting candidates for ACS therapy.

## 5. Conclusions

ACS offers a biologically grounded therapeutic option for OA that can modulate inflammatory and regenerative pathways. Our findings confirm that the standardized IMPACT protocol reliably generates a product with an anti-inflammatory profile, characterized by the consistent presence of IL-1RA and the virtual absence of pro-inflammatory IL-1β, regardless of disease status. However, individual patient characteristics—such as sex, lifestyle, BMI, and age—profoundly influence the composition of ACS.

Specifically, this study identifies a specific molecular signature in ACS from OA patients characterized by a catabolic shift (2.05-fold elevation in MMP-2, 1.64-fold in MMP-3) concurrent with reduced anabolic capacity (0.77-fold TGF-β1), which persists after adjusting for age and sex. These findings, derived from rigorous multiple linear regression analyses, demonstrate that OA pathophysiology—rather than demographic factors—is the primary determinant of ACS composition. Additionally, we identified sex-specific regulation of MMP-3, with males exhibiting 1.75-fold higher levels independent of disease status, highlighting the importance of considering biological sex in ACS therapy and biomarker research.

These findings suggest that a “one-size-fits-all” approach may have limitations. Consequently, while our analyses provide robust evidence for disease-related molecular alterations, the exploratory lifestyle-stratified findings currently only generate hypotheses, and they underscore the need for further studies to investigate whether patient stratification based on these molecular profiles can improve treatment outcomes. IMPACT ACS should be considered as a promising tool whose effectiveness can be optimized through a thorough understanding of the patient’s biological status and rigorous consideration of disease-related molecular signatures.

## Figures and Tables

**Table 1:** Effects shown as fold-changes from log_10_-transformed regression models, adjusted for age and sex. # For MMP-3, males exhibited 1.75-fold higher concentrations than females (*p* < 0.001). ‡ n = 61 due to 9 samples below detection limit. § TGF-β2: Parametric assumptions violated despite transformation (Shapiro–Wilk W = 0.53, *p* < 0.0001); non-parametric Mann–Whitney U test confirmed no significant difference. *** *p* < 0.001, * *p* < 0.05, ns = not significant. VIF < 1.3 for all predictors; all normality tests passed except TGF-β2.

Biomarker	Function	OA Effect (Fold-Change)	95% CI	*p* Value	Age *p* Value	Sex *p* Value	Model R^2^ (adj.)	N
MMP-2	Pro-inflammatory Collagenase	2.05-fold (+105%)	1.56 to 2.71	<0.001 ***	0.325 ns	0.471 ns	0.389	65
MMP-3	Pro-inflammatory Stromelysin	1.64-fold (+64%)	1.23 to 2.18	0.001 ***	0.744 ns	<0.001 ***#	0.371	65
TGF-β1	Anti-inflammatory Anabolic	0.77-fold (−23%)	0.62 to 0.95	0.017 *	0.125 ns	0.084 ns	0.179	61 ‡
TGF-β2	Anti-inflammatory Anabolic	0.96-fold (−4%)	N/A §	0.886 ns	0.141 ns	0.143 ns	0.039	64
TGF-β3	Anti-inflammatory Anabolic	0.89-fold (−11%)	0.78 to 1.03	0.111 ns	0.061 ns	0.252 ns	0.127	61 ‡

## Data Availability

The data presented in this study are available on request from the corresponding author due to privacy, legal, and ethical restrictions.

## Abbreviations

The following abbreviations are used in this manuscript:

ACS	autologous conditioned serum
OA	osteoarthritis
EVs	extracellular vesicles
PRP	platelet-rich plasma
WBS	whole blood serum
IL	interleukin
TNF-α	tumor necrosis factor alpha
MMP	matrix metalloproteinase
ECM	extracellular matrix
